# Food Items

**Published:** 1858-08

**Authors:** 


### FOOD ITEMS.

EVERY hour's exposure to the light, after an Irish potato has been dug from where it grew, deteriorates its quality.

EGGS, when put in water, will, if good, invariably swim with the large end upwards; if not, they are bad.

MRS. HORACE MANN has written a book, entitled, *Christianity in the Kitchen.*

GLASS WARE will be bright and clear if washed in cold water.

WHITE BEANS, at a dollar a bushel, are a more profitable crop than wheat, at a dollar and a quarter a bushel; and, at the same time, make one of the cheapest and most nutritious articles of food we can use.

FOR preparing *Pickles*, cold vinegar should be used; a small piece of alum in each jar makes them firm and crisp.

HOMINY, plain, cheap, healthful, and savory, if boiled one hour, and then enveloped with a blanket until cool, is said to be cooked as thoroughly as if boiled as usual, all day.

TURNIPS are among the least nutritious of all food, nearly ninety per cent being waste; this bulk in the stomach satisfies hunger, while it affords very little nutriment; and as an over-supply of nutriment, eating too much, kills three out of four prematurely, turnips are an advisable article of diet to those who like them, and experience no discomfort after eating them in moderation; while the large amount of waste, by the distension which it occasions, stimulates intestinal action, and thus tends to remove constipation. For these reasons, boiled turnips and brown bread should be largely used, if they agree with them, by invalids and sedentary persons.

